# Job Crafting as a Mediator between Work Engagement and Wellbeing Outcomes: A Time-Lagged Study

**DOI:** 10.3390/ijerph16081376

**Published:** 2019-04-17

**Authors:** Enrique Robledo, Salvatore Zappalà, Gabriela Topa

**Affiliations:** 1 International School of Doctorate, The National Distance Education University (UNED), 28040 Madrid, Spain; pateralmi@gmail.com; 2 Department of Psychology, University of Bologna, 40126 Bologna, Italy; salvatore.zappala@unibo.it; 3 Department of Human Resource Management and Psychology, Financial University under the Government of the Russian Federation, 125993 Moscow, Russia; 4 Department of Social and Organizational Psychology, The National Distance Education University (UNED), 28040 Madrid, Spain

**Keywords:** job crafting, job demands, job resources, work engagement, wellbeing, job performance

## Abstract

This time-lagged study, using the framework of the JD-R model, tested the mediating role of job crafting measuring: at T1, work engagement, workaholism and emotional exhaustion; at T2, job crafting; and, at T3, flourishing, job performance and job satisfaction. Respondents were 443 Spanish employees working in different companies. Results show that job crafting mediates the relationship between work engagement and some of its outcomes (job performance and flourishing). In particular, the job crafting component ‘increasing structural job resources’ mediates the positive effect of work engagement on flourishing and job performance, and the job crafting component ‘increasing challenging demands’ mediates the positive effect of work engagement on job performance. No job crafting mediation is found between work engagement and job satisfaction.

## 1. Introduction

Organizations have recently been more and more aware of the importance of employees’ wellbeing, because this may have positive results for companies and for society, with a clear impact also on public health. Several concepts have been studied by occupational psychology in this regard: work engagement, burnout, stress, job performance and so on. Job crafting is a concept that, although still in its infancy [1], has been developed to better understand the virtuous cycle of employee wellbeing and positive organizational results.

This study contributes to the body of job crafting research with a time-lagged analysis of the relationship between several variables of the Job Demand-Resources (JD-R) model, which is the theoretical framework used in this paper, and some individual outcomes. Following the main principles of the JD-R, we argue that certain aspects of engagement and burnout (such as work engagement, workaholism and exhaustion), measured at T1, will lead to outcomes at T3, particularly flourishing, job satisfaction and job performance. Taking into account job crafting, and the four components of job crafting proposed by Tims, Bakker and Derks [2], at an intermediate time (T2), we will test the mediation processes between the variables at T1 and T3.

This study addresses two gaps in the current job crafting literature. Firstly, many studies investigate work engagement as a consequence of job crafting, while far fewer studies consider engagement as an antecedent of job crafting. Secondly, the few studies that examined job crafting as a consequence of engagement did not go further into considering the effects of this relationship. In this study, we hypothesize that this association would have an effect on wellbeing and job performance that is different for the different components of job crafting.

In particular, the core contribution of this study is the analysis of the differential effects of the job crafting components on engagement outcomes and in the mediation processes between engagement and outcomes. Additionally, this study makes other contributions to the field of job crafting research: (a) it looks at job crafting as a consequence of engagement, and not the other way around, as most previous research has; (b) it provides a time-lagged analysis of the JD-R model with quite a comprehensive set of variables; (c) more importantly, we integrate previous studies that showed that work engagement promotes wellbeing and contributes to organizational development, by proving that job crafting is one of the mechanisms that explains that relationship.

In the following sections, we briefly describe the JD-R theoretical framework and how the two focal variables of the study, engagement and job crafting, fit with it. Then, we present the rationale of our hypotheses, describing why engagement, workaholism and emotional exhaustion (as antecedent variables) are related to job crafting (the mediator) and to our dependent variables (flourishing, job satisfaction and job performance). Then, we discuss the main target of this study, which is hypothesizing and testing the differential effects that job crafting components have on the individual outcomes examined here.

### 1.1. Job Crafting and Work Engagement in the JD-R Model

The term job crafting was coined by Wrzeniewski and Dutton [3] as the physical and cognitive changes individuals make in their task or relationship boundaries. It concerns the proactive changes in job design that are not negotiated with organizations, and probably not even noticed by the manager [4]. According to Wrzeniewski and Dutton [3], employees can change how work is conceptualized and carried out (i.e., changing task boundaries), how often and with whom they interact at work (i.e., changing relationship boundaries), and how they cognitively ascribe meaning and significance to their work (i.e., changing meaning).

The integration of job crafting in the JD-R model was proposed by Tims and Bakker [4]. In the JD-R model, all job characteristics can be categorized into two types: job demands or job resources. Job demands refer to all aspects of the job that require sustained physical and/or psychological (cognitive and emotional) effort or skills. Therefore, job demands are associated with certain physiological or psychological costs. Job resources refer to those aspects of the job that are either/or functional in achieving work goals, reduce job demands and the associated physiological and psychological costs, and stimulate personal growth, learning, and development [5].

Job crafting in the JD-R model framework is defined as the changes employees introduce in their job demands and job resources to better meet their personal abilities and needs [2]. This conceptualization does not consider the cognitive dimension of job crafting and focuses only on real changes that employees make in their jobs. This is the conceptualization followed in this paper.

According to Tims, Bakker and Derks [2], job crafting can take the form of four types of behavior: (a) increasing social job resources, (b) increasing structural job resources, (c) increasing challenging job demands; and (d) decreasing hindering job demands. This factor structure for job crafting has also been found when job crafting is measured on a daily basis [6,7].

Work engagement is defined as a positive, fulfilling and work-related state of mind that is characterized by vigor, dedication and absorption [8]. The vigor component refers to how stimulating, energetic and worth devoting time to the work is perceived by the workers to be. Dedication reflects a significant and meaningful pursuit. Absorption is the component that describes when the workers are fully concentrated and immersed into the task.

Work engagement, extensively studied in the JD-R model, is influenced by job resources such as autonomy, feedback, social support and skill variety, as reported in many research papers and meta-analyses (e.g., [9,10]). This relationship is explained by arguing that job resources promote employees’ extrinsic motivation (in other words, they increase their interest in achieving work goals) and intrinsic motivation (fostering employees’ desire for growth, learning and development), which, in turn, affects work engagement. Moreover, in the present study, work engagement can be considered to be a wellbeing indicator. Positive psychology has stated that individual wellbeing can be conceptualized both as hedonic and eudaimonic wellbeing [11] Hedonic wellbeing refers to the affective and cognitive components of satisfaction assessment, like job satisfaction and flourishing, whereas eudaimonic wellbeing refers to the individual’s psychological and social functioning [12].

Psychological wellbeing consists of self-realization, but also of social integration, contribution, and actualization, among other factors [13,14]. Based on this approach, eudaimonic wellbeing is connected to the social dimension, and would include employees’ work engagement, and other behaviors positively oriented to other people in the work environment or to their organization [15]. 

### 1.2. Job Crafting as a Consequence of Wellbeing State

In this section, we discuss how job crafting is related to employee wellbeing state measured by the level of work engagement, emotional exhaustion and workaholism of the employee. We first analyze the relationship of job crafting with positive wellbeing state measured by work engagement. Then, we take into account the relationship between job crafting and the negative state variables of emotional exhaustion and workaholism.

There is an extensive literature about the impact of job crafting on work engagement, with job crafting considered a predictor of work engagement [16,17,18,19]. However, here we take a different position, and we believe that a reversed causal positive relationship is possible [20], and that work engagement may also promote job crafting. It is, in fact, likely that employees that feel motivated and enthusiastic and, therefore, engaged with their job will be more likely to be proactive and to craft their job [7]. Also, job crafting behaviors are dependent, both in intensity and in typology, on task-contextual and personal factors, like type of job and type of personality. Job crafting has been positively related to proactive personality [16], and according to Roczniewska and Bakker [21], personality plays an important role when choosing how to craft one’s job. In this sense, it seems reasonable to think that not only personality, but also wellbeing state, such as work engagement, could influence the intensity and type of job crafting.

However, not many studies have empirically proved this causal relationship. Harju, Hakanen and Schaufeli [22] found a cross-lagged effect over time between work engagement and the two job crafting components of increasing social and structural resources. In Hakanen, Peeters and Schaufeli [23], this causal relationship was tested longitudinally. In a sample of 1877 Finnish dentists, they found that work engagement positively predicted increasing social and structural resources and challenging demands, and negatively predicted decreasing hindering job demands. Based on the theory and previous research described above, we hypothesize that:

**H1:**  *Work engagement at T1 is an antecedent of job crafting at T2*.

One question is if job crafting, beyond being influenced by a positive wellbeing state of mind (work engagement), is also influenced by other negative wellbeing states of mind, such as emotional exhaustion or work addiction.

Emotional exhaustion is the clearest manifestation of burnout, a psychological syndrome in response to job stressors, which is characterized by emotional exhaustion, cynicism, and reduced professional efficacy [24]. Workaholics are, instead, employees that work excessively and compulsively, investing their resources continuously in work, often at the expense of their private life and regardless of whether they fail or succeed [25].

Some studies have observed a negative relationship between burnout and job crafting components. Petrou, Demerouti and Schaufeli [26] found that emotional exhaustion predicted decreasing hindering job demands, and vice versa. The only study we have found that analyses the relationship between workaholism and job crafting is Hakanen, Peeters and Schaufeli [23], where they also studied the influence of burnout on job crafting components. They found different relationships between workaholism, burnout and specific job crafting components in a longitudinal sample of Finnish dentists. In particular, they found that workaholism positively predicted increasing structural resources and challenging demands, and that burnout positively predicted decreasing hindering job demands and negatively predicted increasing structural resources.

We also expect a stronger causal relationship between work engagement and job crafting than between emotional exhaustion, workaholism and job crafting. One of the reasons is that, as explained above, engagement has been proved to be related to all components of job crafting, while emotional exhaustion and workaholism are only related to some of them. Another reason is that, according to Bakker and Demerouti’s [27] model, job crafting and work engagement are concepts included in the JD-R motivational process line (in which high job resources lead to positive organizational outcomes) and not in the health impairment line (where chronic high job demands lead to strain and health problems), where it is more reasonable to locate the main influence of emotional exhaustion and workaholism. Therefore, we forecast the following:

**H2:**  *Work engagement at T1 is a stronger predictor of job crafting at T2 than workaholism and emotional exhaustion*.

### 1.3. Job Crafting Mediation between Work Engagement and its Outcomes

Studies and meta-analyses have shown that work engagement has many consequences with respect to task performance and contextual performance, which are positive for workers and their organizations [9,10]. It is also believed that job crafting behaviors are mainly associated with positive outcomes, since proactive employees, capable of modifying their working environment, are also more likely to contribute positively to the organization [5]. In addition, job crafting behaviors, by improving person–job fit, put workers in a position to achieve better performance and also to have a better wellbeing condition [28]. This positive relationship was also observed in a quasi-experimental study, conducted with teachers, in which it is shown that a job crafting intervention had positive effects on employee wellbeing [29].

The question is whether these two lines of influence, from engagement to positive outcomes and from job crafting to positive outcomes, are somehow linked. The research so far has studied the mechanism by which job crafting creates more engagement and other concurrent positive outcomes, but there is not, thus far, any research that analyses whether a particular engagement state can build on job crafting behaviors to produce positive personal and organizational outcomes.

Thus, based on the JD-R model and on empirical studies, we forecast that engaged employees will craft their job by introducing changes in resources and demands, thus creating better conditions to reach higher levels of positive outcomes. In another way, there is an indirect effect of work engagement leading to positive outcomes through job crafting. This leads us to formulate the following:

**H3:**  *Job crafting, at T2, mediates the effect of work engagement at T1, on job performance, job satisfaction and flourishing, at T3*.

### 1.4. Differential Effects of Job Crafting Components

Many authors agree that job crafting components behave differently and have underlying differential processes with the variables with which they have been related, and this could be a reason for some inconsistencies observed in job crafting studies.

In particular, inconsistencies have been observed in studies relating such components with engagement. We mentioned above how in the study by Hakanen, Peeters and Schaufeli [23], all job crafting components were positively related to engagement, except decreasing hindering job demands, which was negatively related. Instead, Sakuraya et al. [30] report that decreasing job hindering demands is not related to work engagement, while the other components are.

There are even some authors that split the job crafting components into two groups with different properties. This is the case for Tims, Bakker and Derks [31]. They define expansive components (increasing structural resources, increasing social resources and increasing challenging demands) vs. hindering demands components (decreasing hindering job demands). Demerouti [1] outlines expansive job crafting, which is described as including seeking resources and new challenges and coping-related job crafting, comprising decreasing negative aspects of the job.

The differential effect of job crafting components on consequences generated by wok engagement has not yet been tested. However, considering that job crafting components seem to have different relationships with engagement, we forecast that job crafting components will also have different effects with job performance, job satisfaction and flourishing, and, in particular, that the component decreasing hindering job demands will not mediate between engagement and outcomes, while the others components will have a mediating effect.

**H4:**  *Job crafting components behave differently in the mediation process; in particular, decreasing hindering job demands at T2 will not mediate between engagement at T1 and positive outcomes at T3, while the other three components will mediate*.

## 2. Materials and Methods

### 2.1. Participants

To alleviate common method variance concerns, data were collected in three rounds (from now on, T1, T2, and T3), with a 4-month time lag [32]. A sample of 443 Spanish white-collar employees, working in health and education (32, 5%), industry (8, 9%), banking (7, 8%), public administration (4, 2%) and other services, answered three questionnaires. At T1, engagement, workaholism and exhaustion were measured; at T2, we measured job crafting; and at T3, we measured job performance, job satisfaction and flourishing.

Women comprised 63% of the sample. The average age was 41. Average tenure was 11.64 years; 73% of respondents held a university degree, and 46% were managers. Gender, age, tenure, education and organizational level were used as control variables; all respondents were white-collar workers, and therefore we did not control for type of job.

### 2.2. Measures

Work engagement was measured using the Spanish validated version of the Utrecht Work Engagement Scale (UWES-9) [33]. This scale is the reduced version of the 17-item UWES. It contains 9 items in three subscales: Vigor (e.g., “At my work I feel bursting with energy”), dedication (e.g., “I am enthusiastic about my job”), and absorption (e.g., “I feel happy when I am working intensively”). Answers are given on a five-point scale, ranging for (1) never to (5) very often.

Workaholism was measured using the Spanish validated version of the Dutch Work Addiction Scale (DUWAS) [34]. The scale consists of 10 items in two subscales: working excessively (WE; e.g., “I seem to be in a hurry and racing around the clock”) and working compulsively (WC; e.g., “it is important to me to work hard even when I do not enjoy what I am doing”). Answers are given on a five-point scale, ranging from (1) never to (5) very often.

Emotional exhaustion was measured using 5 items from the Maslach Burnout Inventory-General Survey (MBI-GS) [35], translated into Spanish for this study. An example of one item is: “I am emotionally exhausted by my job”. Answers are given on a five-point scale, ranging from (1) never to (5) very often.

Job crafting was measured using the Job Crafting scale developed by Tims, Bakker and Derks [36], validated for the Spanish language [37]. It contains 21 items in four subscales: increasing social job resources (ISR), increasing structural job resources (ISJR), increasing challenging job demands (ICJD) and decreasing hindering job demands (DJD). Examples are: “I ask my supervisor to coach me” (ISR); “I try to develop my capabilities” (ISJR); “when an interesting project comes along, I offer myself proactively as project co-worker” (ICJD), and “I make sure that my work is mentally less intense” (DJD). Answers follow a five-point scale, ranging from (1) never to (5) very often.

Job Satisfaction was measured by 4 items of the Brief Affective Job Satisfaction Scale (BIAJS) [38], validated for the Spanish language [39]. An example of one item is: “I really enjoy my job”. Answers are given on a five-point scale, ranging from (1) never to (5) very often.

Flourishing was assessed using the Spanish version (FS-SV) [40] of Diener et al.’s Flourishing Scale [41]. The scale assesses major aspects of social-psychological functioning, as having good social relationships, a purposeful and meaningful life, and being interested in one’s activities. An example of one item is: “I am optimistic about my future”. Answers are given on a five-point scale, ranging from (1) never to (5) very often.

Job performance was measured by the 7-item In-Role Behavior Scale (IRB) developed by Williams and Anderson [42]. The original version in English has been already used in the Spanish language in a previous study [43]. A sample item is: “fulfills responsibilities specified in the job description”. Answers are given on a five-point scale, ranging from (1) never to (5) very often. Items 6 and 7 are reversed items.

#### 2.2.1. Procedure

The present study was approved by the Ethical Committee of the UNED in 2018.

The HR department of companies that had worked with us in previous studies were contacted and invited to participate in an online study about career planning. Companies that agreed to participate distributed to their workers a link to the online questionnaire built with the tool Google Forms. Worker participation was voluntary and confidential. The first part of the questionnaire comprised an informed consent form. Respondents were then informed of the voluntary and anonymous nature of their participation, of the aims of the research project and of the fact that they were free to abandon the study at any time without penalty. To protect respondents’ anonymity, no personal information was collected (such as, for instance, personal or firm emails, IP, organization or department membership). Participants created a personal code that allowed us to match the answers that each participant gave to the three waves of the survey.

#### 2.2.2. Data Analysis

Hypotheses 1 and 2 were tested through a hierarchical regression methodology. To test these two hypotheses, which concern the impact of work engagement, as well workaholism and emotional exhaustion, on job crafting, we used the global indicator of job crafting, because this makes it easier to examine the order of causality and the relative impact across independent variables. We tested Hypotheses 3 and 4 in two steps using structural equation modeling (SEM) analysis with the AMOS software package [44]: in the first step, we tested the measurement model, and in the second step, we tested the structural paths. To test the fit of alternative models to the data we used the traditional chi-square, the normed chi-square (CMIN/DF), the Root Mean Square Error Approximation (RMSEA) and the comparative fit index (CFI). The values considered to be a good fit of the model to the data are RMSEA< 0.08 [45], CMIN/DF < 0.5 [46] and CFI > 0.90 [47]. For these two hypotheses, we used the global indicator and the four components of job crafting, because we are interested in the differential effect of such components in the mediation processes.

## 3. Results

Table 1 shows correlations between all variables and their components, along with Cronbach alphas. All variables have a high reliability, with all Cronbach alphas being well above 0.70. One of the relevant results from Table 1 is that there is no significant correlation between workaholism and work engagement or between workaholism and the general job crafting indicator. However, workaholism is positively correlated with emotional exhaustion (*r* = 0.43), and emotional exhaustion is negatively correlated with engagement (*r* = −0.54). These results are similar to those observed by Schaufeli et al. [48]. Work engagement is well related with the global indicator and all the components of job crafting (from *r* = −0.18 to *r* = 0.54), and, similarly, to the other outcome variables.

To test Hypothesis 1 and 2, a hierarchical regression of job crafting at T2 was run with respect to the T1 variables. In step 1, the control variables were introduced. In step 2, all T1 variables were introduced at the same time. The results in Table 2 show that engagement is clearly an antecedent of job crafting (Beta = 0.52, *p* < 0.01) (which confirms Hypothesis 1) and that neither workaholism nor emotional exhaustion have a significant influence on job crafting. Therefore, work engagement is clearly a stronger predictor of job crafting than workaholism and emotional exhaustion (thus confirming Hypothesis 2).

To explore H3, a set of hierarchical regressions was run, with job performance, job satisfaction and flourishing regressed with respect to the T3, T2 and T1 variables. In step 1, the same control variables as before were introduced. In step 2, T1 variables were introduced. In step 3, job crafting was introduced. In step 4, the other T3 variables were introduced. Results are also shown in Table 2.

Looking at the impact of job crafting, measured at T2, on outcome variables measured at T3 (step 3), we note that job crafting is a predictor of job performance (Beta = 0.23, *p* < 0.01) and flourishing (Beta = 0.14, *p* < 0.05), but not of job satisfaction. In addition, we note that when we introduce job crafting as a predictor of job performance, the influence of work engagement disappears (from Beta = 0.20, *p* < 0.01, to Beta= −0.08, *p* = non-significant), so it seems that job crafting is a full mediator between engagement and job performance. For flourishing, however, introducing job crafting does not fully takes out the influence of engagement (whose Beta goes from 0.50, *p* < 0.01, to 0.43, *p* < 0.01), so it seems that job crafting is a partial mediator between engagement and flourishing. In this way, Hypothesis 3 is partially accepted, as job crafting mediates the relationship between engagement and wellbeing outcomes for job performance and flourishing, but not for job satisfaction.

**Figure 1:**
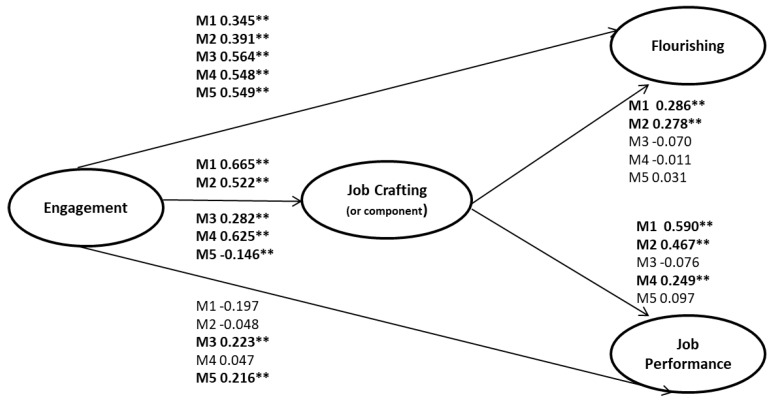
Path modeling of alternative models (Betas, ** *p* < 0.01).

Job satisfaction is predicted by engagement and emotional exhaustion, and does not have a direct relationship with job crafting. For this reason, we tested the mediation of job crafting and its components (Hypothesis 4) only on job performance and flourishing (see Figure 1) using a structural equation modeling approach. We first tested the measurement model, which showed a good fit to the data: χ2 (543) = 1454, CMIN/DF = 2.582, CFI = 0.902, RMSEA = 0.06.

To test the path model, we tested the significance of the paths of 5 different models. In M1, we tested the mediation of the global job crafting measurement; in M2, we tested the mediation of increasing structural resources component; in M3, we tested the mediation of increasing social resources component; in M4, we tested the mediation of increasing challenging demands; and in M5, we tested the mediation of the decreasing job demands component.

All models have a good goodness of fit, as shown in Table 3. Path results are reported in Figure 1. The scores of the relationship of work engagement with flourishing and job performance, reported in Figure 1, take into account the mediating effect of job crafting (and its components), and thus represent the indirect effects.

As a summary of Figure 1, and related to Hypothesis 3 and 4, our results suggest that: (a) both global job crafting and increasing structural resources are partial mediators between work engagement and flourishing; (b) global job crafting, increasing structural resources and increasing challenging demands are full mediators between work engagement and job performance; (c) no mediation is observed with decreasing hindering job demands and increasing social resources; (d) decreasing hindering job demands is negatively related to engagement, while all other components are positively related to work engagement.

In other words, job crafting partially mediates between engagement and flourishing, and the mediation is due to the increase in structural resources. Job crafting also fully mediates between engagement and job performance, and the mechanisms are due to the increase of structural resources and the increase of challenging demands.

Therefore, we partially accept Hypothesis 4, because decreasing job demands, as expected, is not a mechanism in the mediation process, but we have not found the expected mediation of increasing social resources.

## 4. Discussion

This time-lagged study demonstrates first that worker engagement state will determine the level of job crafting behaviors that employees will deploy in the future: the higher the engagement, the higher the level of job crafting behaviors.

Secondly, this study shows that work engagement is a stronger predictor of job crafting behaviors than workaholism and burnout, the effects of which are not comparable with engagement, in the sense that they do not affect job crafting behaviors at all. Therefore, we can conclude that job crafting, as a global concept, is triggered by positive (engagement) and not by negative (emotional exhaustion and workaholism) states of mind.

These results seem to be in line with the principles of the JD-R model [5]. Hence, in line with Bakker and Demerouti [49], there is a motivational process, to which engagement and job crafting contribute, and a health impairment process, which sees its main contributions from emotional exhaustion and workaholism. The health impairment line in this study, and in line with the JD-R model [5], is linked with the motivational line through the negative correlation between emotional exhaustion and engagement.

Thirdly, the study shows that there is an indirect effect of work engagement on job performance and flourishing through job crafting. This result, which was expected according to theory, had not been observed before now in previous studies. This not only means that engaged employees are more prone to developing job crafting behaviors, but also that these job crafting behaviors contribute to improved job performance and social-psychological wellbeing. Some studies have already demonstrated that job crafting increases engagement; thus, taking into account our results, we can anticipate a virtuous gain loop: ‘engagement leads to job crafting, which leads to more engagement, which leads to more job crafting, and so on’. This is the idea of the spiral gain previously proposed by Schaufeli, Bakker and Rhenen [25]: “initial work engagement predicts an increase in job resources, which, in its turn, further increases work engagement”.

Fourthly, we confirmed that the component decreasing hindering job demands has a different effect compared to the other job crafting components. This differential effect is found in many research studies, and even in the original paper by Tims et al. [36] on the development and validation of the Job Crafting Scale. In our study, decreasing hindering job demands does not correlate with the rest of the job crafting components, or with job performance and flourishing, and it is negatively correlated with engagement and job satisfaction and positively correlated with emotional exhaustion. It also seems clear that decreasing job demands is not a mechanism in the mediation between engagement and outcomes.

The interpretation is that decreasing hindering job demands can be seen as something positive (I want to obtain the best performance so I prioritize my tasks and ignore other requests) or it can be interpreted as something negative (I do not like my job, so I try to do as little as possible of what is expected of me). This positive or negative characterization may depend on the sample or on the specific context, but on the whole it will not have a relevant impact on engagement and positive outcomes. This dependency on context may explain the results observed by Dierdorff and Jensen [50], which conclude that job crafting might have dysfunctional consequences for performance-related outcomes under certain conditions of task and social context. Another explanation is that decreasing job demands might be too broad a component that might contain multiple concepts. Something similar was stated by Nielsen and Abildgaard [51], who found two types of decreasing job demands (hindering and social) that were differently related to other psychosocial variables.

One unexpected result was the lack of influence of the increasing social resources component in the mediational process. This is not in line with the finding of Hakanen, Peeters and Schaufeli [23], but is close to the result observed by van Windergen, Bakkers and Derks [29], who found that increasing social resources was not affected by an intervention to increase job crafting.

What is clear in our research is that increasing structural job resources and increasing challenging job demands are the two strongest job crafting components in the relationship between work engagement and outcomes. In fact, they seem to be the most productive mechanisms to leverage the effect of job crafting in order for an engaged workforce to achieve positive outcomes.

### 4.1. Limitations and Practical Implications

The limitations in this study come mainly from the type of methodology used in the study. A first limitation is the time-lagged design. While it provides a more rigorous test for non-spurious associations than cross-sectional studies, and avoids the common method bias, a full longitudinal analysis, collecting measures of work engagement, job crafting and outcomes in the three time periods, would have allowed a more rigorous causal analysis. Another limitation is that job performance is only measured by self-ratings of in-role performance and not by peer or supervisor reports of in-role and extra-role performance. Another limitation could be the four-month time lag between measurements, which could have not been enough to capture some effects over time, for instance the effect of some job crafting components on flourishing or job performance. Another limitation is that we did not compute the percentage of variance that the indirect effect accounts for. This could have given an idea of the strength of the mediation, although we believe that this is not a problem in our study, as we were looking for mediation vs. no mediation effect, rather than the relative strength of mediations. Despite the fact that our participants, and employees in general, are nested in departments and companies, we did not track the organization or the group memberships of our respondents. This decision was based on the desire to guarantee anonymity for the participants, but it entails a loss of information. If we had had access to nested structured data, we could have conducted multilevel analyses accounting for part of variance in our outcomes due to belonging to specific organizations and departments. Given that organizational culture and practices can act as situational constraints on job crafting, further research could be conducted that avoids these limitations. Another limitation of this paper could be related to the conceptual approach followed. The use of just the JD-R model as a theoretical framework is debatable, and it could be interpreted as a limited or partial perspective. It could be advisable to interpret and integrate the results and findings in light of more sound theories like basic need satisfaction [52,53] or the self-concordance model [54]. 

Practical implications concern the positive effects of work engagement. In detail, this study suggests that enhancing work engagement may be an effective way to increase job crafting and prevent poor wellbeing. Organizations should also be aware of the influence of job crafting as a tool for increasing job performance and workers’ wellbeing; they should also promote interventions that foster employees’ proactivity to increase structural resources and challenging demands, which are the two most influential components in the job crafting boosting process.

These findings are not just useful for organizations. Implications for public health may be related to the increase of the wellbeing of an already positive labor workforce. A public health policy that facilitates training and interventions in job crafting could be a powerful tool for increasing job performance. Van Windergen et al. [29] conclude that a job crafting intervention could increase the resource opportunities for professional development. In fact, self-initiated skills development at work is a type of job crafting [55]. Also, Akkermans and Tims [56] state that job crafting mediates the positive relationship between career competencies and career success, measured by both internal and external perceived employability. Linking these studies to the findings in our study, we can conclude that it is important to facilitate self-learning and development activities (increasing structural job resources) that are at the same time challenging (increasing challenging demands), so the effect of engagement on job performance and wellbeing are maximized.

### 4.2. Future Research

Finally, there are some additional side results, non-core for the objectives of the study, but that are worth noting because they open the door for future studies.

Job satisfaction is only predicted by engagement and emotional exhaustion, and not by job crafting. This is in line with Hakanen, Peeters and Schaufeli [23], where job satisfaction was found not to relate to job crafting. It is interesting to see that job satisfaction is not related to job crafting while flourishing is, despite both being wellbeing outcomes of the motivational line. Our interpretation of this is that although both job satisfaction and flourishing are wellbeing variables and outcomes of engagement, flourishing conveys some active role of the employee (“I lead a purposed and meaningful life”, “I actively contribute to the happiness and wellbeing of others”) and not just a passive role, as in the case of job satisfaction; therefore, flourishing is closer to job crafting than job satisfaction is. To interpret this result, further investigation of the role of job satisfaction in the JD-R motivational process and its relationship with job crafting is needed.

The absorption dimension of engagement has a different behavior from vigor and dedication, as it is the only engagement component correlated with workaholism. Therefore, absorption can have a dual interpretation: one positive and close to the concept of flow, and the other negative and closer to the concept of workaholism. This finding could be incorporated to the body of research in work engagement for further analysis.

In the job crafting indicator, there seems to be an influence of the education level, meaning that the higher the level of education, the higher the level of job crafting behaviors deployed. We have not found any study relating job crafting to education level, so further research is needed.

## 5. Conclusions

This study, using a cross lagged design, has showed that job crafting is one of the mechanisms that allows an engaged workforce to achieve positive outcomes, like high job performance and high flourishing. In particular, the job crafting components ‘increasing structural job resources’ and ‘increasing challenging job demands’ seem to be the most productive job crafting mechanisms through which an engaged workforce may achieve positive personal and work outcomes.

## Figures and Tables

**Table 1:** Pearson correlations and reliabilities of the study variables.

	Variables Used in the Study	1	2	3	4	5	6	7	8	9	10	11	12	13	14	15	16
1	Emotional Exhaustion	0.90															
2	Work engagement	−0.54 **	0.93														
3	Vigor	−0.61 **	0.91 **	0.85													
4	Dedication	−0.54 **	0.94 **	0.83 **	0.89												
5	Absorption	−0.31 **	0.87 **	0.66 **	0.74 **	0.79											
6	Workaholism	0.42 **	0.08	−0.06	0.04	0.25 **	0.88										
7	WE	0.45 **	0.07	−0.07	0.03	0.23 **	0.93 **	0.80									
8	WC	0.33 **	0.07	−0.05	0.04	0.23 **	0.91 **	0.69 **	0.77								
9	Job Crafting	−0.14 **	0.44 **	0.41 **	0.43 **	0.36 **	0.12 **	0.10 *	0.12 **	0.79							
10	ISJR	−0.29 **	0.54 **	0.52 **	0.55 **	0.41 **	0.04	0.05	0.02	0.64 **	0.81						
11	DJD	0.20 **	−0.18 **	−0.17 **	−0.18 **	−0.14 **	−0.07	−0.07	−0.05	0.47 **	0.02	0.78					
12	ISR	−0.14 **	0.30 **	0.29 **	0.31 **	0.22 **	0.07	0.04	0.09	0.65 **	0.22 **	0.06	0.76				
13	ICJD	−0.20 **	0.53 **	0.46 **	0.49 **	0.49 **	0.27 **	0.26 **	0.24 **	0.69 **	0.55 **	−0.06	0.29 **	0.83			
14	Job Performance	−0.17 **	0.18 **	0.18 **	0.19 **	0.11 *	−0.13 **	−0.12 **	−0.12 **	0.22 **	0.38 **	0.04	0.04	0.19 **	0.84		
15	Flourishing	−0.39 **	0.48 **	0.54 **	0.47 **	0.30 **	−0.22 **	−0.19 **	−0.22 **	0.30 **	0.47 **	−0.022	0.13 **	0.25 **	0.29 **	0.88	
16	Job Satisfaction	−0.60 **	0.82 **	0.77 **	0.83 **	0.63 **	−0.049	−0.02	−0.06	0.36 **	0.49 **	−0.19 **	0.26 **	0.43 **	0.18 **	0.46 **	0.92

Note: WE = Working excessively; WC = Working compulsively; * *p* < 0.05; ** *p* < 0.01; Cronbach alphas are on the diagonal.

**Table 2:** Multiple regression analysis.

Variables	Job Crafting		Job Satisfaction				Job Performance				Flourishing			
	Step1	Step2	Step1	Step2	Step3	Step4	Step1	Step2	Step3	Step4	Step1	Step2	Step3	Step4
Control variables														
Age	−0.03	−0.04	−0.02	−0.04	−0.04	−0.04	−0.02	−0.04	−0.03	−0.03	0.00	−0.03	−0.02	−0.02
Tenure	−0.03	−0.04	0.03	0.00	0.00	0.01	0.00	−0.02	−0.01	−0.01	0.03	−0.01	0.00	0.00
Gender	0.04	0.03	0.00	0.03	0.03	0.03	0.02	0.03	0.02	0.03	0.00	0.02	0.02	0.02
Education	0.13 **	0.11 **	0.01	−0.01	−0.01	−0.01	−0.08	−0.07	−0.10	−0.08	−0.04	−0.04	−0.06	−0.04
Org Level	0.05	0.08	−0.11 *	−0.04	−0.04	−0.04	−0.08	−0.07	−0.08	−0.07	−0.06	−0.03	−0.04	−0.02
T1 variables														
Engagement		0.52 **		0.70 **	0.68 **	0.66 **		0.20 **	0.08	−0.01		0.50 **	0.43 **	0.36 **
Workaholism		0.02		−0.02	−0.02	0.00		−0.17 **	−0.18 *	−0.10 *		−0.26 **	−0.26 **	−0.22 **
Emotional Exhaustion		0.11		−0.22 **	−0.23 **	−0.21 **		0.03	0.01	0.03		−0.02	−0.04	−0.03
T2 variable														
Job Crafting					0.03	0.03			0.23 **	0.20 **			0.14 **	0.10 *
T3 variables														
Job Satisfaction										0.00				0.08
Job Performance						0.00								0.15 **
Flourishing						0.04				0.20 **				
R^2^	0.02	0.24	0.01	0.71	0.71	0.71	0.01	0.06	0.10	0.13	0.01	0.32	0.33	0.36
R^2^ Change	0.02	0.22 **	0.01	0.70**	0.00	0.00	0.01	0.05 **	0.04 **	0.03	0.01	0.31 **	0.01	0.03

Note: * *p* < 0.05, ** *p* < 0.01.

**Table 3:** Fit indices of the alternative models.

Model	χ2 (df)	*p*	CMIN/DF	CFI	RMSEA
M1—Global Job crafting mediation	1408 (544)	0.000	2.588	0.902	0.060
M2—Increasing structural resources mediation	802 (243)	0.000	3.304	0.916	0.072
M3—Increasing social resources mediation	829 (264)	0.000	3.140	0.910	0.070
M4—Increasing challenging demands mediation	731 (220)	0.000	3.325	0.916	0.072
M5—Decreasing hindering demands mediation	652 (200)	0.000	3.261	0.910	0.072
